# Health-Related Quality of Life of HIV-Positive and HIV-Negative Pregnant Women in an Impoverished Area: Cross-sectional Study

**DOI:** 10.2196/29906

**Published:** 2022-04-05

**Authors:** Shuiling Qu, Ailing Wang, Xiaoyan Wang, Yehuan Yang, Xiaoping Pan, Tong Zhang

**Affiliations:** 1 Chinese Center for Disease Control and Prevention Beijing China; 2 National Center for Women and Children's Health Chinese Center for Disease Control and Prevention Beijing China; 3 Capital Institute of Pediatrics Beijing China

**Keywords:** health-related quality of life, EQ-5D-3L, HIV, impoverished area, public health, pregnant women, depression, anxiety

## Abstract

**Background:**

Liangshan prefecture of Sichuan province was an impoverished mountainous area in China, where the annual number of HIV-positive pregnant women accounted for approximately 10% of China’s total population in the decades before 2020. In general, pregnant women living here are likely to be physically and mentally different from those in other places.

**Objective:**

This study aims to explore the health-related quality of life (HRQoL) of pregnant women living with HIV in an impoverished area.

**Methods:**

From December 2018 to January 2019, HIV-positive and HIV-negative parturients within 18 months after delivery were recruited in Liangshan Prefecture, Sichuan Province. Questionnaires were designed to collect their demographic data, while the EuroQol 5-Dimension, 3-Level questionnaire was used to measure their HRQoL when they were in the second trimester from 4 to 6 months of pregnancy, and their quantitative health scores were converted to corresponding healthy utility values by using the Chinese Utility Value Integral System (time trade-off coefficient).

**Results:**

A total of 250 pregnant women (133 HIV-positive and 117 HIV-negative) were enrolled in the study. Among them, 55 (41.35%) and 75 (64.10%) of HIV-positive and HIV-negative pregnant women self-reported full health (healthy state 11111), respectively. The median health utility value of the 250 pregnant women was 0.961 (IQR –0.046 to 0.961), and those of the HIV-positive and HIV-negative pregnant women were 0.875 (0.424-0.961) and 0.961 (IQR –0.046 to 0.961), respectively. We observed a significant difference only in the dimension of anxiety or depression between the two groups (*P*=.002) and no significant difference in the distribution of health utility indices between the two groups in terms of maternal age, education level, occupation, annual household income, prenatal care visits, family size, and medical insurance category. Multivariate ordinal logistic regression analysis showed that age (odds ratio [OR] 0.62, *P*<.05) and prenatal care visit (OR 0.29, *P*<.01) were independent risk factors for health status.

**Conclusions:**

Most pregnant women self-reported satisfactory HRQoL in this impoverished mountainous area. HIV-negative pregnant women had an edge over HIV-positive pregnant women, and there were significant differences in anxiety or depression dimensions between the two groups.

## Introduction

Generally, pregnant women have a poorer health-related quality of life (HRQoL) owing to the impact of pregnancy on their physiological and mental health [1]. Apart from limited physical activities in early pregnancy, most pregnant women are likely to experience pregnancy syndromes such as nausea, vomiting, dizziness, depression, nervousness, and anxiety throughout their pregnancy [2]. Moreover, compared with HIV-negative pregnant women, HIV-positive pregnant women have also been reported to have poorer overall physique [3], higher susceptibility to depression [3], and poorer mental health [4]. As evidenced by previous research, educational background, number of children, pregnancy symptoms, and occupation are all key factors influencing the HRQoL of pregnant women [2,5]. Labor losses and indirect costs for HIV and HRQoL are strongly associated with severity [6]. HIV/AIDS is also associated with a significant economic burden for caregivers living with HIV [7]. Nowadays, with 1.3 million (range 970,000 to 1.6 million) HIV-positive pregnant women worldwide in 2020 [8], HIV continues to be a major global public health issue. Accordingly, improvement of patient care—by evaluating HRQoL in HIV-positive pregnant women—is of great importance to inform decision-making, resource allocation, and health policy formulation.

HRQoL could be measured by using not only generic scales such as the World Health Organization Quality of Life-100 (WHOHRQOL-100), the EuroQoL 5-Dimension (EQ-5D) questionnaire, and the 36-Item Short Form survey (SF-36), but also specific scales such as the Medical Outcomes Study HIV Health Survey (MOS-HIV). The EQ-5D is used as a multi-attribute utility instrument for measuring HRQoL, which comprises a short, cognitively undemanding questionnaire that takes only a few minutes to complete [9,10].

In China, the general rate of mother-to-child transmission (MTCT) of HIV decreased from 34.8% before 2005 to 3.6% in 2020 over the years [11]. In Liangshan Prefecture, the highest HIV-affected epidemic region in Sichuan Province, China, where the annual number of local HIV-positive pregnant women is estimated to be 10% of China’s total in recent decades, the rate of MTCT of HIV was 4.3%. The per capita GDP of Liangshan Prefecture, an impoverished mountainous area, is far lower than that of Sichuan Province [12]. Previous studies on HIV-positive pregnant women in Liangshan focused on sexual behaviors [13], changing modes of HIV transmission [14], perceptions of social norms [15], HIV prevalence [16], etc. Hence, this cross-sectional study aimed to explore the HRQoL of HIV-positive pregnant women in the Liangshan Prefecture, Sichuan Province.

## Methods

### Study Design and Participants

From December 2018 to January 2019, in Liangshan Prefecture, Sichuan Province, HIV-positive and -negative parturients within 18 months after delivery were recruited to the clinic for a questionnaire survey one by one. The questionnaire contained questions about demographic data including age, marital status, education level, and annual household income. The HRQoL of pregnant women was evaluated using the EQ-5D-3L. The inclusion criteria for participant recruitment were being registered residents of Liangshan Prefecture, having given birth in Liangshan prefecture, and having no severe mental or neurological disease.

Previous studies have shown that most HIV-positive women in Liangshan Prefecture are from the Yi minority, with a lower education level, and speak in Yi language [17,18]. In this study, we sought local doctors to help participants interpret the questionnaire and translate it to us.

Since this was a cross-sectional study, the following formula was used to estimate the sample size:




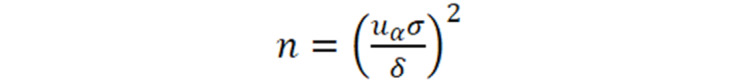




As a part of study on cost-effectiveness analysis, the personal indirect costs were set as the SD at CNY 500 (US $78.56), the allowable error (δ) was set at CNY 100 (US $15.71), and (α=.05) was 1.96. The minimum required sample size of HIV-positive pregnant women was 96.

### Assessment

The EQ-5D-3L questionnaire was composed of five dimensions: mobility (MO), self-care (SC), usual activities (UA), pain or discomfort (PD), and anxiety or depression (AD); each dimension was divided into three levels: no problems, some problems, and extreme problems. The participants were asked to indicate their healthy state by ticking the box next to the most appropriate statement for each of the 5 dimensions. The healthy state could be either converted into corresponding healthy utility value by using the Chinese Utility Value Integral System (time trade-off coefficient; Table 1) [19] or graded into 4 healthy state levels in accordance with the EQ-5D-3L [20]. The formula for healthy utility value is as follows: F=1 – C - MO- SC – UA – PD – AD - N3, where N3 equals 0.000 or 0.022 if any dimension is at level 3.

Steps for grading a healthy state are as follows: first, the distance between each healthy state and full health is calculated (11111; for example, the distance between 12321 and 11111 is equal to 1 + 2 + 3 + 2 + 1 – 1 – 1 – 1 – 1 – 1 = 4). The mild healthy state “2” indicates that the distance is between 1 and 4, no dimension is at level 3, and there are at most three dimensions at level 2. The severe healthy state “4” implies that the distance is between 7 and 9, no dimension is at level 1, and at least two dimensions are at level 3. The others are in a moderate healthy state “3.”

**Table 1:** Chinese Utility Value Integral System (time trade-off coefficient) for the EuroQol 5-Dimension questionnaire.

Dimension	Mobility				Self-care				Usual activities				Pain or discomfort				Anxiety or depression				C		N3
Level	1	2	3	1		2	3	1		2	3	1		2	3	1		2	3				
Coefficient	0	0.099	0.246	0		0.105	0.208	0		0.074	0.193	0		0.092	0.236	0		0.086	0.205	0.039		0.022/0.000	

### Statistical Analysis

The chi-square test or the Fisher exact test was used to test the difference in the proportion of education level, occupation, and health insurance category between HIV-positive and HIV-negative pregnant women. Moreover, the Fisher exact test was applied to test the differences between the two groups under various health dimensions and factors influencing HRQoL, respectively. Multivariate ordinal logistic regression analysis for categorical data analysis was used for analysis of risk factors for health status. R packages vcd and MASS were used for the chi-square test, the Fisher exact test, and multivariate ordinal logistic regression analysis. The acquired data were then compared using the 2-tailed *t* test, with the level of statistical significance determined at α=.05.

### Ethical Procedures

This study has been approved by the Ethics Review Committee of the National Center for Women and Children’s Health, Chinese Center for Disease Control and Prevention (FY2018-06) and that of the Chinese Center for Disease Control and Prevention (201922).

## Results

### Baseline Characteristics of the Recruited Subjects

In total, 250 pregnant women (133 HIV-positive and 117 HIV-negative) were recruited in this study (Table 2). The average age was 30.52 (range 18-53) years, with a significant difference between HIV-positive (mean 32.37, SD 5.62 years) and HIV-negative (mean 28.41, SD 7.07 years) pregnant women (*t*_248_=4.85, *P*<.001). 

**Table 2:** Baseline characteristics of pregnant women.

Characteristics			HIV-positive pregnant women, n (%)		HIV-negative pregnant women, n (%)		Chi square (*df*)			*P* value	
**Age (years)**								7.709 (1)			.005
	18-35	86 (64.66)		95 (81.20)							
	35-53	47 (35.34)		22 (18.80)							
**Education level**								1.692 (1)			.19
	Illiterate	114 (85.72)		92 (78.63)							
	Primary or junior high school	19 (14.28)		25 (21.37)							
**Occupation**								0.032 (1)			.86
	Farmer	118 (88.72)		102 (87.18)							
	Migrant worker	15 (11.28)		15 (12.82)							
**Annual household income (CNY)^a^**								1.692 (1)			.19
	<8000	70 (52.63)		51 (43.59)							
	≥8000	63 (47.37)		66 (56.41)							
**Prenatal care** **visits**								3.797 (3)			.28
	≤2	48 (36.09)		56 (47.86)							
	3	34 (25.56)		25 (21.37)							
	4	20 (15.04)		16 (13.68)							
	≥5	31 (23.31)		20 (17.09)							
**Family size (persons)**								0.265 (2)			.88
	3	18 (13.53)		16 (13.68)							
	4-5	70 (52.63)		58 (49.57)							
	≥6	45 (33.84)		43 (36.75)							
**Medical insurance**								0.005 (1)			.95
	Insured	126 (94.74)		112 (95.73)							
	Uninsured	7 (5.26)		5 (4.27)							

^a^1CNY=US $0.16.

### Health Utility Measurements

The median health utility value was 0.961 (IQR –0.046 to 0.961), and those of the HIV-positive and HIV-negative pregnant women were 0.875 (IQR 0.424-0.961) and 0.961 (IQR –0.046 to 0.961), respectively. A significant difference was only found on the AD dimension between the two groups (*P*=.002) and not on other dimensions. Regarding SC, 98% of pregnant women had “no problems,” 5 (1.88%) had “some problems,” and 0 had “serious problems.” Approximately 90% and 85% of the pregnant women had “no problems” in the MO and UA dimensions, respectively, whereas for the PD dimension, 75% of the pregnant women had “no problems” and 24% of them had “some problems” (Table 3).

In total, 130 (52.00%) of pregnant women were in full health (healthy state, 11111), including 55 (41.35%) HIV-positive pregnant woman and 75 (64.10%) HIV-negative pregnant women. Only 1 HIV-negative pregnant woman transitioned to grade 4 owing to the presence of other serious diseases after further inquiry. Consequently, this subject was excluded from the subsequent survey of health grade comparison between groups, per the chi-square test. A significant difference in health status between HIV-positive and HIV-negative pregnant women was observed (χ^2^_2_=12.5, *P*=.002). Furthermore, HIV-positive pregnant women had a relatively poorer health status than HIV-negative pregnant women (Table 4).

**Table 3:** Health status of pregnant women under different EuroQol 5-Dimension dimensions.

Dimensions			HIV-positive pregnant women (n=133), n (%)		HIV-negative pregnant women (n=117), n (%)		Fisher exact test (*P* value)	
**Mobility**								.45
	1	119 (89.47)		107 (91.45)				
	2	14 (10.53)		9 (7.69)				
	3	0 (0)		1 (0.86)				
**Self-care**								<.001
	1	130 (97.74)		115 (98.29)				
	2	3 (2.26)		2 (1.71)				
**Usual activities**								.14
	1	109 (81.95)		104 (88.89)				
	2	23 (17.29)		11 (9.40)				
	3	1 (0.75)		2 (1.71)				
**Pain or discomfort**								.53
	1	98 (73.68)		91 (77.78)				
	2	34 (25.56)		24 (20.51)				
	3	1 (0.75)		2 (1.71)				
**Anxiety or depression**								.002
	1	77 (57.89)		90 (76.92)				
	2	52 (39.1)		22 (18.80)				
	3	4 (3.01)		5 (4.28)				

**Table 4:** Grading of healthy states of pregnant women (N=250).

Grading of healthy states	HIV-positive pregnant women (n=133), n (%)	HIV-negative pregnant women (n=117), n (%)	Total, n
Full health	55 (41.35)	75 (64.10)	130
Mild health	45 (33.83)	22 (18.80)	67
Moderate health	33 (24.81)	19 (16.24)	52
Severe health	0 (0)	1 (0.86)	1

### Analysis of Factors Influencing Health Utility

Based on the results of the chi-square or the Fisher exact test, no significant difference was observed in pregnant women’s age, education level, occupation, annual household income, prenatal care visits, family size, and type of health insurance category on the distribution of health grade. However, a lower proportion (25.00%) of the uninsured than insured pregnant women was observed in grade 1 (Table 5).

Multivariate ordinal logistic regression analysis showed that age (odds ratio [OR] 0.62, *P*<.05) and prenatal care visits (OR 0.29, *P*<.01) were independent risk factors for health status (Table 6).

**Table 5:** Independent analysis of health status and relevant factors.

Characteristics			Health grade n (%)									Chi-square (*df*)				*P* value	
			1		2		3		4^a^								
**Age (years)**													5.170 (2)				.75
	20-35	102 (56.35)		65 (35.91)		14 (7.73)		0 (0)									
	35-53	28 (40.58)		35 (50.72)		5 (7.25)		1 (1.45)									
**Education level**													N/A^b^				.90^c^
	Illiterate	107 (51.94)		83 (40.29)		15 (7.28)		1 (0.49)									
	Primary or junior high school	23 (52.27)		17 (38.64)		4 (9.09)		0 (0)									
**Occupation**													N/A				.57^c^
	Farmer	113 (51.36)		90 (40.91)		16 (7.27)		1 (0.45)									
	Migrant worker	17 (56.67)		10 (33.33)		3 (10.00)		0 (0)									
**Annual household income (CNY)^d^**													1.557 (2)				.46
	<8000	62 (51.24)		52 (42.98)		7 (5.79)		0 (0)									
	≥8000	68 (52.71)		48 (37.21)		12 (9.30)		1 (0.78)									
**Prenatal care visits**													N/A				.16^c^
	≤2	61 (58.65)		36 (34.62)		6 (5.77)		1 (0.96)									
	3	29 (49.15)		26 (44.07)		4 (6.78)		0 (0)									
	4	20 (55.56)		15 (41.67)		1 (2.78)		0 (0)									
	≥5	20 (39.22)		23 (45.10)		8 (15.69)		0 (0)									
**Family size (person)**													N/A				.88^c^
	3	19 (55.88)		12 (35.29)		3 (8.82)		0 (0)									
	4-5	69 (53.91)		50 (39.06)		9 (7.03)		0 (0)									
	≥6	42 (47.73)		38 (43.18)		7 (7.95)		1 (1.14)									
**Health insurance**													N/A				.05^c^
	Insured	127 (53.36)		91 (38.24)		19 (7.98)		1 (0.42)									
	Uninsured	3 (25.00)		9 (75.00)		0 (0)		0 (0)									

^a^N/A: not applicable.

^b^*P* values obtained using the Fisher exact test.

^c^In view of the distribution and professional judgment, the sample of health grade 4 was deleted during statistical analysis.

^d^1CNY=US $0.16.

**Table 6:** Multivariate ordinal logistic regression analysis of risk factors for health status.

Factor	B (SE)	*t* test (df)	*P* value	OR (95% CI)
Intercept 1|2	1.16 (0.39)	2.98 (1)	<.01	N/A^a^
Intercept 2|3	3.63 (0.46)	7.86 (1)	<.001	N/A
Age	0.62 (0.28)	2.23 (248)	<.05	0.62 (0.16-1.08)
Prenatal care visit	0.29 (0.11)	2.61 (248)	<.01	0.29 (0.11-0.47)

## Discussion

### Principal Findings

Our results indicate that 41.35% of HIV-positive pregnant women and 64.10% HIV-negative pregnant women self-reported full health. The median health utility value of the 250 pregnant women was 0.961. A significant difference was only observed in the dimension of anxiety or depression between the two groups.

This serious ceiling effect [21] may be the negative impact of the sensitivity of health measurement, which differed from the discovery of a good health utility measurement for HIV-positive pregnant women in Yunnan Province [22]. The difference may be due to the following reasons. First, 89.01% of households in Yunnan Province had an annual income of more than 10,000 CNY (US $1571.29), which was higher than that of the participants in this study [22]. The average annual food expenditure of a family and the annual household income in this study were CNY 4000 (US $628.52) and CNY 8000 (US $1257.03), which translated into an Engel coefficient of 57.14%. The Engel coefficient indicated that the people were in the stage of barely meeting daily needs in 2017 according to the Engel law. Second, participants in Yunnan Province had better education than participants in this study. Third, most of the local residents are very sturdy as they live in a mountainous area of Liangshan. If the HIV-positive pregnant women in this study were compared with HIV/AIDS cases, the HRQoL was superior to that of those without antiviral treatment (0.801) [23] and those with antiviral treatment (0.82) [24].

As reported herein, health utility value of HIV-negative pregnant women was higher than that of HIV-positive pregnant women. Based on the analysis on the five dimensions in the EQ-5D-3L, a significant difference was found only in the AD dimension between the HIV-positive and HIV-negative pregnant women, but not in the MO, SC, UA, and PD dimensions. Similarly, it was reported [25,26] that HIV-positive patients had a higher risk of developing depression, which suggested that HIV-positive pregnant women may have psychological burden, and psychological intervention could exert a significant positive effect. Theoretically, the status of health may be affected by the degree of education, occupation, annual household income, and other factors, but this study was not able to validate this point just yet. Nevertheless, HRQoL was still found to be significantly positively correlated with annual household income, degree of education, and occupation [27]. The difference may be explained by sampling. As described above, Liangshan Prefecture was an impoverished area, with a homogenous income source structure, education, and occupation. In this study, age, and prenatal care visits were independent risk factors for health status. Mobile health or SMS text messaging could be used for improving the quality of antenatal care [28,29].

### Limitations

This study still has the following limitations. First, it was designed as a retrospective analysis, serving as a part of the health economics evaluation of the prevention of MTCT of HIV in Liangshan Prefecture. There may be a recall bias among the pregnant women when recalling their HRQoL during their second trimester of pregnancy. Studies on general HRQoL assessment with the EQ-5D-3L appear largely free of recall bias within follow-up visits of 2-12 months [30], and agreement of HRQoL determined using the EQ-5D-3L between conventional (1 week) and retrospective change (3 months later) is fair [31], indicating that the recall bias could be accepted. Second, there may be a considerable selection bias due to the low feasibility of a random sampling owing to the particularity of HIV-positive pregnant women themselves.

In addition, HIV-positive pregnant women surveyed in this study experienced more anxiety or depression during pregnancy than HIV-negative ones. Further communication revealed that the anxiety was generated from the fear of MTCT of HIV. Hence, there is a need for further emphasis, research, and intervention to improve the mental health of HIV-positive pregnant women.

### Conclusions

An interactive voice response tool may be a choice for people living with HIV, and the higher usage of the tool showed greater improvements in quality of life [32]. Mobile phones were found both to be acceptable and feasible in the collection of maternal and child health data from women living with HIV in South Africa. Accordingly, publicity and education are necessary to achieve full awareness of the prevention of MTCT of HIV to increase the confidence of the population group.

## Abbreviations

AD: anxiety or depression
EQ-5D-3L: EuroQol 5-Dimension, 3-Level
HRQoL: health-related quality of life
MO: mobility
MOS-HIV: Medical Outcomes Study HIV Health Survey
MTCT: mother-to-child transmission
PD: pain or discomfort
SC: self-care
SF-36: 36-Item Short Form survey
TTO: time trade-off
UA: usual activities
WHOHRQOL-100: World Health Organization Quality of Life-100
